# Dynamic DNA methylation in tea plants and its association with changes in gene expression under salt and alkali stress

**DOI:** 10.1186/s43897-025-00189-5

**Published:** 2026-02-05

**Authors:** Xiangrui Kong, Hongli Cao, Dandan Lou, Chuan Yue, Ruiyang Shan, Shiqin Zheng, Aodi Han, Xingtan Zhang, Changsong Chen, Weilong Kong

**Affiliations:** 1 Tea Research Institute, Fujian Academy of Agricultural Sciences, Fuzhou, 350013 Fujian China; 2 Integrative Science Center of Germplasm Creation in Western China (CHONGQING) Science City, College of Food Science, Southwest University, Chongqing, 400715 China; 3 Key Laboratory of Horticultural Plant Biology, Ministry of Education, National Key Laboratory for Germplasm Innovation & Utilization of Horticultural Crops, College of Horticulture and Forestry Sciences, Huazhong Agricultural University, Wuhan, 430070 China; 4 National Key Laboratory for Tropical Crop Breeding, Shenzhen Branch, Guangdong Laboratory for Lingnan Modern Agriculture, Genome Analysis Laboratory of the Ministry of Agriculture, Agricultural Genomics Institute at Shenzhen, Chinese Academy of Agricultural Sciences, Shenzhen, Guangzhou, 518120 China

Salt and alkali stresses induce gene expression changes in tea plant (*Camellia sinensis* (L.) O. Kuntze), subsequently affecting plant growth and development (Wan et al. 2024; Zhang et al. 2017, 2023). Recent advances in phenomics and transcriptomics have elucidated physiological and gene expression alterations in tea seedlings under saline stress (Wan et al. 2018, 2020, 2024; Zhang et al. 2017, 2023). However, the epigenetic regulatory mechanisms underlying these gene expression changes remain unclear. Therefore, this study investigated the dynamics of transcriptional and 5mC methylation modifications under salt and alkali stress.

In RNA sequencing (RNA-seq), one-year-old tea plant seedlings were treated with salt (200 mM NaCl) or alkali (150 mM NaHCO_3_) solution for 0 (0d, before stress treatment), 3 (3d), and 7 (7d) days. Significant wilting of young leaves emerged after the salt and alkali stress treatments, with wilting severity, peroxidase (POD), and superoxide dismutase (SOD) content increasing over time (Fig. 1A**, **S1). The study generated 99.79 Gb of clean RNA-seq data from 15 samples (three biological replicates per treatment), achieving an average read mapping rate of 93.59% (Table S1 and Fig. S2, S3). RNA-seq analysis identified a > fourfold increase in the number of differentially expressed genes (DEGs) in samples treated for 7 d (NC-7d and NHC-7d) compared to the control (CK-0d), relative to those treated for 3 d (NC-3d and NHC-3d). This finding indicated that extended stress durations induce broader gene expression changes (Fig. 1B). Correspondingly, plants exhibited a more severe wilting phenotype at day 7 than at day 3 post-treatment (Fig. 1A). Kyoto Encyclopedia of Genes and Genomes (KEGG) functional annotation of DEGs revealed that down-regulated DEGs (Down-DEGs) in all groups were significantly enriched in phenylpropanoid and flavonoid biosynthesis pathways (Fig. 1C) as well as photosynthesis and DNA replication (Fig. 1C and D), indicating that salt and alkali stress affect flavonoid and catechin synthesis and photosynthetic systems in tea plants. Conversely, up-regulated DEGs (Up-DEGs) showed significant enrichment in diterpenoid and brassinosteroid biosynthesis pathways (Fig. 1C), alongside several established abiotic stress-related pathways, including MAPK signaling, plant hormone signal transduction, signal transduction, and environmental adaptation (Fig. 1D).

**Fig. 1:**
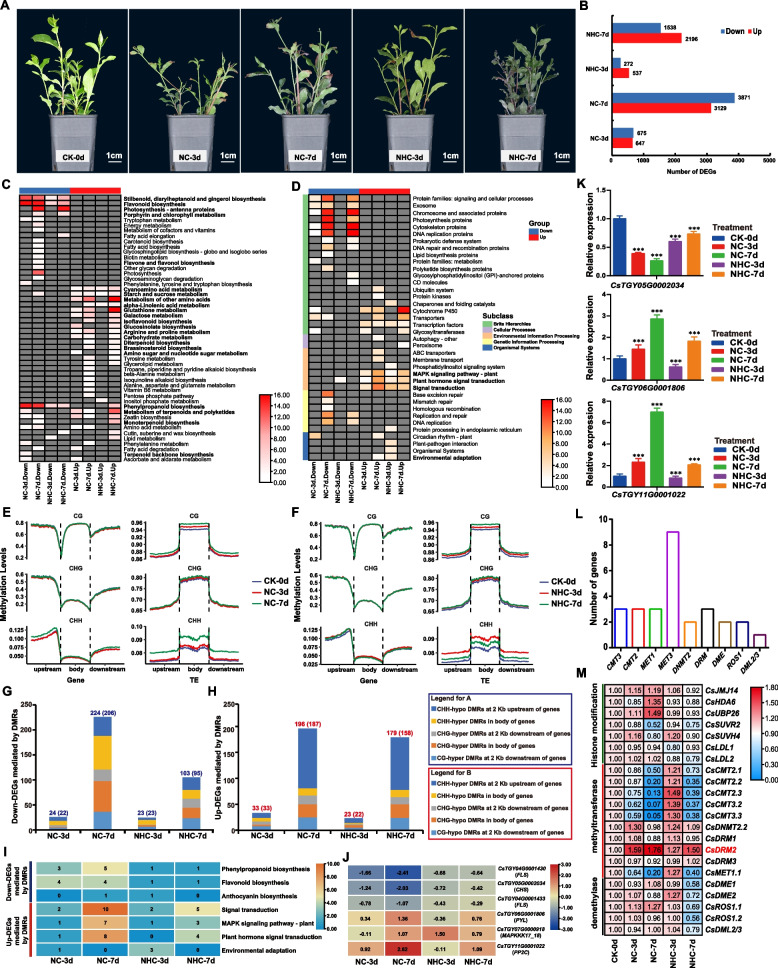
**A** Morphological changes in tea plant seedlings under salt and alkali stress (CK, NC, and NHC represent the control, NaCl, and NaHCO_3_ treatments, respectively.). Each treatment included three independent biological replicates. **B** Number of differentially expressed genes (DEGs) in NC-3d vs. CK-0d (NC-3d), NC-7d vs. CK-0d (NC-7d), NHC-3d vs. CK-0d (NHC-3d), and NHC-7d vs. CK-0d (NHC-7d). Up and Down represent up- and down-regulated DEGs, respectively. **C **and** D** KEGG annotation results (*P* value < 0.05) of different DEGs (the abbreviation for difference comparisons is the same as Fig. 1B). **E **and** F** Methylation level changes in the body and upstream and downstream 2 Kb regions of genes and transposons (TEs) under salt stress and alkali stress (the abbreviation for treatments is the same as Fig. 1A). **G **and** H** Number of Down-DEGs mediated and up-DEGs by five different DMRs in NC-3d, NC-7d, NHC-3d, and NHC-7d (the abbreviation for difference comparisons is the same as Fig. 1B). **I** KEGG annotation of Down-DEGs or Up-DEGs mediated by five different DMRs (the abbreviation for difference comparisons is the same as Fig. 1B). **J** Fold change (Log_2_ processed) in the expression of several DMR-mediated DEGs in the flavonoid synthesis pathway and the MAPK signaling pathway (the abbreviation for difference comparisons is the same as Fig. 1B). **K** Relative gene expression results from qRT‒PCR analysis of *CsTGY05G0002034* (*CHS*), *CsTGY06G0001806* (*PYL*), and *CsTGY11G0001022* (*PP2C*). *** indicates *p* < 0.001 (paired t test) (the abbreviation for treatments is the same as Fig. 1A). **L** Number of genes associated with methylation and demethylation in tea plant genome. **M** Gene expression changes in methylation and demethylation-related genes under salt and alkali stress (the abbreviation for treatments is the same as Fig. 1A)

Venn diagram analysis classified the DEGs into two categories: core DEGs, defined as those exhibiting consistent up-regulation or down-regulation at day 3 and day 7 post-treatment relative to day 0; and period-specific DEGs, defined as those differentially expressed only at either day 3 or day 7 post-treatment relative to day 0. Under alkali stress, this analysis identified 6610 period-specific DEGs and 1098 core DEGs (Up: 545 + Down: 553) (Fig. S4A). Similarly, under salt stress, 3837 period-specific DEGs and 331 core DEGs (Up: 166 + Down: 165) were detected (Fig. S4B). Further analysis of these four sets of core DEGs identified 413 Down-core DEGs and 483 Up-core DEGs under alkali stress, 25 Down-core DEGs and 104 Up-core DEGs under salt stress, and 140 Down-core DEGs and 62 Up-core DEGs that were common to both salt and alkali stress conditions (Fig. S4C). KEGG results showed that these core DEGs participated in different pathways, with a correlation between DEG quantity and pathway involvement (Fig. S5). Four established abiotic stress-related pathways—signal transduction, plant hormone signal transduction, carbohydrate metabolism, and starch and sucrose metabolism—were consistently enriched across all six core DEG sets, though with varying numbers of genes involved (Fig. S5).

In addition, we performed transcription factor (TF) identification analysis in these six core DEG sets and identified 10, 56, 1, 14, 5, and 7 TF-encoding core DEGs in the NC.Core.Down, NC.Core.Up, NHC.Core.Down, NHC.Core.Up, NC and NHC.Core.Down, as well as NC and NHC.Core.Up DEG sets (Fig. S4C and Fig. S6). In previous studies, several of these TFs were experimentally validated to demonstrate functional effects under salt and alkali stress (Fig. S4D), including *WRKY33* and *ERF98* (Jiang and Deyholos 2009; Zhang et al. 2012). Additionally, we identified five core DEGs in the ABA signal transduction pathway, two core DEGs in the MAPK cascade pathway, nine core DEGs in the Ca^2+^ signal transduction pathway, and 14 core DEGs encoding different transporter proteins (Fig. S4E). These findings demonstrated that maintaining homeostasis under salt and alkali stress involves multiple transporter proteins.

Considering the highly heterozygous nature of the tea plant genome, we developed a novel approach combining whole-genome bisulfite sequencing (WGBS-seq) and resequencing (Fig. S7), enabling DNA methylation sequencing on the same samples previously analyzed by RNA-seq (Table S2). WGBS-seq analysis revealed CG-, CHG-, and CHH-type methylation levels of 89.51%–90.88%, 69.37%–70.65%, and 7.80%–8.58% (Fig. S8), respectively, differing from previous findings (84.88%, 68.41%, and 12.60% for CG, CHG, and CHH methylation) (Kong et al. 2023). This variation primarily results from our improved method, which accounts for false-positive methylation quantification caused by genome-wide C/T heterozygous sites. The analysis revealed that methylation levels of all types were significantly elevated in treated samples compared to untreated samples (Fig. S8), indicating that salt and alkali stress induced substantial changes in methylation modifications. Further comparison of methylation levels in the body, upstream, and downstream regions of genes and transposable elements (TEs) revealed significant changes in the upstream and downstream regions of genes, as well as in the body and flanking regions of TEs (Fig. 1E and F). These results suggested that tea plants may regulate gene expression under salt and alkali stress by modifying the methylation levels of genes and TEs. Differential methylation analysis and annotation results showed that hypermethylated differentially methylated positions (hyper-DMPs) and hypermethylated differentially methylated regions (hyper-DMRs) significantly outnumbered hypomethylated DMPs (hypo-DMPs) and hypomethylated DMRs (hypo-DMRs) (Fig. S9A). The DMRs were predominantly found in distal intergenic and promoter regions, with a minor fraction overlapping gene coding regions (Fig. S9B).

To investigate the effects of DNA methylation on gene expression, this study analyzed the relationships between mCG, mCHG, and mCHH levels in the gene region, at 2 Kb upstream and downstream regions of genes and their expression levels. The analysis revealed a negative correlation between gene expression levels and methylation levels in the 2 Kb downstream region for mCG, in the gene body for mCHG, in the 2 Kb downstream region for mCHG, and in the gene body for mCHH (Fig. S10). Notably, a positive correlation was observed between methylation levels in the 2 Kb upstream region of the gene for mCHH and gene expression levels (Fig. S10), aligning with findings in moso bamboo (Ding et al. 2022) and apple (Xu et al. 2018). Based on these correlations, the study identified DEGs mediated by DMRs in specific regions: 2 Kb downstream region for mCG, gene body for mCHG, 2 Kb downstream region for mCHG, 2 Kb upstream region for mCHH, and gene body for mCHH. The analysis identified 22, 206, 23, and 95 non-redundant Down-DEGs mediated by these five different DMRs in NC-3d vs. CK-0d (NC-3d), NC-7d vs. CK-0d (NC-7d), NHC-3d vs. CK-0d (NHC-3d), and NHC-7d vs. CK-0d (NHC-7d), respectively (Fig. 1G and Table S3). Correspondingly, 33, 187, 22, and 158 non-redundant Up-DEGs mediated by five different DMRs in NC-3d, NC-7d, NHC-3d, and NHC-7d, respectively (Fig. 1H). KEGG annotation highlighted the impact of differential methylation modification on expression changes of genes related to flavonoid synthesis and stress response pathways (Fig. 1I), including *FLS* (*flavonol synthase*) and *CHS* (*chalcone synthase*) relating to flavonoid synthesis in the Down-DEGs mediated by DMRs and *PYL* (*abscisic acid receptor PYR/PYL family*) (Cutler et al. 2010; Ren et al. 2022), *MAPKKK17/18* (*mitogen-activated protein kinase kinase kinase 17/18*) (Chardin et al. 2017; Zhang et al. 2018), and *PP2C* (*protein phosphatase 2C*) (Cutler et al. 2010; Park et al. 2009; Schweighofer et al. 2004) in the MAPK signaling pathway in the Up-DEGs mediated by DMRs (Fig. 1J, S11, K). Additionally, three core DEGs, namely, *WRKY33*, *MPK3*, and *ABC transporter*, associated with both salt and alkali stress were found to be affected by methylation modification changes (Table S4).

Through BLASTP and the HMM algorithm analysis of the monoploid TGY genome, genes encoding demethylases, methyltransferases, and histone modifications were identified (Fig. 1L and M). RNA-seq and qRT‒PCR analysis (primers in Table S5) revealed that the up-regulated expression of a single methylation-related gene (*CsDRM2*) effectively explained the increase in methylation levels under stress (Fig. 1M, S12), suggesting that *CsDRM2* up-regulation might be the primary cause of increased methylation levels under stress. However, considering that expression changes of methylation-related genes at certain time points can explain methylation level changes, the increase in genome-wide weighted methylation also might result from the combined effects of multiple methylation-related genes.

In summary, these findings enhance the understanding of epigenetic mechanisms in tea plants under abiotic stress and identify important gene targets for future breeding improvement through epigenetic variation manipulation.

## Supplementary Information

Supplementary Material 1. Supplementary Materials and Methods.

Supplementary Material 2. Supplementary Figures.

Supplementary Material 3. Supplementary Tables.

## Data Availability

The raw sequence data reported in this paper have been deposited in the Genome Sequence Archive at the National Genomics Data Center (GSA: CRA022976) and are publicly accessible at https://ngdc.cncb.ac.cn/gsa.
