# Supplementary material for: Dynamic DNA methylation in tea plants and its association with changes in gene expression under salt and alkali stress
Source: Mol Hortic. 2026 Feb 5;6:10. doi: 10.1186/s43897-025-00189-5 (PMC12874681; doi:10.1186/s43897-025-00189-5)
Supplement: Supplementary file 1 — Supplementary Material 1. Supplementary Materials and Methods. [file 43897_2025_189_MOESM1_ESM.docx]

**Dynamic DNA methylation in tea** **plants and its association with changes in gene expression under salt and alkali stress**

Xiangrui Kong ^1+^, Hongli Cao ^2+^, Dandan Lou ^3^, Chuan Yue ^2^, Ruiyang Shan ^1^, Shiqin Zheng ^1^, Aodi Han ^1^, Xingtan Zhang ^4*^, Changsong Chen ^1*^, and Weilong Kong ^4*^

**^1^** Tea Research Institute, Fujian Academy of Agricultural Sciences, Fuzhou, Fujian 350013, China;

**^2^** Integrative Science Center of Germplasm Creation in Western China (CHONGQING) Science City, College of Food Science, Southwest University, Chongqing, China;

**^3^** Key Laboratory of Horticultural Plant Biology, Ministry of Education, National Key Laboratory for Germplasm Innovation & Utilization of Horticultural Crops, College of Horticulture and Forestry Sciences, Huazhong Agricultural University, Wuhan 430070, China;

**^4^** National Key Laboratory for Tropical Crop Breeding, Shenzhen Branch, Guangdong Laboratory for Lingnan Modern Agriculture, Genome Analysis Laboratory of the Ministry of Agriculture, Agricultural Genomics Institute at Shenzhen, Chinese Academy of Agricultural Sciences, Shenzhen, Guangzhou 518120, China;

**Corresponding author**,

Email:

[zhangxingtan@caas.cn](mailto:zhangxingtan@caas.cn);

[ccs6536597@163.com](mailto:ccs6536597@163.com);

Weilong.Kong@whu.edu.cn

**Materials and methods**

**Plant materials and treatments**

All stress experiments were conducted at the tea plant farm of Anxi County (25.05°N, 118.18°E), Quanzhou city, Fujian Province. We used field-grown one-year-old tea plant seedlings (*C. sinensis* var. *sinensis* cv. ‘Tieguanyin’) as experimental materials and subjected them to salt and alkali stress via irrigation with 200 mM NaCl and 150 mM NaHCO_3_ (once every two days) (Wan et al. 2024; Zhang et al. 2023). Young leaves (one bud and two leaves) were then collected at 10 AM before treatment (0 d), on the 3rd day of treatment (3 d), and on the 5th day of treatment (5 d). All the collected samples were immediately immersed in liquid nitrogen and stored at -80 °C. In this study, three independent biological replicates were prepared for each treatment for RNA-seq and WGBS-seq sequencing.

**Measurement of important physiological indexes**

The POD and SOD contents of the samples were determined by spectrophotometry using a UV–Vis spectrophotometer (UV-1800, Shimadzu) (Wan et al. 2024). The samples used for POD and SOD assessment were processed with nitrogen blue tetrazole (NBT) and guaiacol, respectively.

**RNA-seq analysis**

Total RNA from all stress-treated and untreated samples was extracted using the Tiangen Total RNA Extraction Kit (DP441, Beijing, China), and cDNA sequencing libraries were constructed using the QIAquick PCR Kit (Qiagen, Venlo, The Netherlands). The cDNA libraries were subsequently sequenced on the DNBSEQ-T7 platform (BGI, Shenzhen, China), generating 150 bp paired-end reads.

All the raw reads were filtered using fastp with the default parameters (Chen et al. 2018), and the clean reads were mapped onto the monoploid TGY reference (Zhang et al. 2021) genome using HISAT2 (Kim et al. 2015). The mapped clean reads were quantified on the basis of the number of fragments per kilobase of transcripts per million mapped reads (FPKM) using featureCounts (Liao et al. 2014). DEGs with corrected P values (FDRs) < 0.05 and fold changes ≥ 2.0 were identified by DESeq2 (Cao et al. 2024). KEGG enrichment analysis of the DEGs was performed using TBtools (Chen et al. 2020).

**WGBS-seq analysis**

Total DNA extraction and WGBS-seq library construction for all the samples were performed as previously described (Kong et al. 2023). The WGBS-seq raw reads were filtered using fastp with the following parameters: -5, -3, and --cut_right for --cut_window_size 4 --cut_mean_quality 20, --cut_window_size 4, and --detect_adapter_for_pe -q 15 -u 40 -e 20 -n 5 -l 30 -p -P 20 (Kong et al. 2023). Then, the clean WGBS-seq reads were mapped onto the monoploid TGY reference (Zhang et al. 2021) using Bismark (Krueger and Andrews 2011) with the default parameters, and bam files from Bismark were subsequently sorted using SAMtools. We retained only unique reads with mapping quality values greater than 20 in the bam file through SAMtools (-q 20 -F 4 -F 256) for the next step of the quantitative analysis of the methylation levels. Finally, we calculated the methylation levels of the genome-wide methylation sites via the calmeth program in BatMeth2 (Zhou et al. 2019) with the following parameters: -Q 20, --remove_dup, --coverage 4, and -nC 1.

In WGBS-seq, sodium bisulfite converts unmethylated C bases to U bases, whereas methylated C bases remain unchanged under sodium bisulfite treatment. After PCR amplification, U bases are converted to T bases. Thus, the level of methylation can be determined by calculating the ratio of C to T (Guo et al. 2014). However, for highly heterozygous woody plants such as tea plants, many C/T heterozygous single-nucleotide polymorphisms (SNPs) can affect the accurate assessment of methylation levels. Therefore, we first conducted deep genome-wide resequencing (WGR-seq) to identify genome-wide C/T heterozygous SNPs and then excluded these C/T heterozygous SNPs from subsequent methylation quantification and comparative analyses (**Fig. 2A**). For WGR-seq, DNA was extracted using the DNeasy Plant Mini Kit (Qiagen, Beijing, China), and 5 μg of genomic DNA was used to construct a library with an insert size of 500 bp. The library was then sequenced with paired-end 150 bp reads on the DNBSEQ-T7 platform (BGI, Shenzhen, China). The raw reads were filtered using fastp with the default parameters (Chen et al. 2018), and the clean reads were mapped onto the monoploid TGY reference (Zhang et al. 2021) genome using BWA (Li and Durbin 2009) with the default parameters. We retained only unique reads with mapping quality values greater than 10 in the bam file through SAMtools (-q 10 -F 4 -F 256) for all SNP calling via gatk (McKenna et al. 2010) HaplotypeCaller with the default parameters.

**I****dentification of DMRs and DMPs**

We identified genome-wide DMRs using MethylKit (Akalin et al. 2012) with the following parameters: --window 1200 —step 600 —mincov 4; DMRs of CG and CHG with methylation differences ≥ 0.2 and P values < 0.05; and DMRs of CHH with methylation differences ≥ 0.1 and P values < 0.05. Similarly, we identified DMPs by MethylKit (Akalin et al. 2012) with the following parameters: --window 600--step 200 —mincov 4; DMPs of CG and CHG with methylation differences ≥ 0.2 and P values < 0.05; and DMPs of CHH with methylation differences ≥ 0.1 and P values < 0.05. In this study, DEGs with DMPs or their upstream 2 Kb or downstream 500 bp regions overlapping with DMRs were considered that regulated by differential DNA methylation.

**Genome-wide identification of DNA methylases and demethylases, as well as histone modification genes related to DNA methylation**

In *Arabidopsis*, several key genes that influence the overall level of genome-wide methylation, including genes encoding methyltransferases such as *MET1*, *DRM2*, *CMT2*, and *CMT3*, as well as genes encoding demethylases, namely, *DME*, *ROS1*, and *DML2/3,* have been identified via molecular studies (Law and Jacobsen 2010; Matzke et al. 2015; Wang et al. 2019). In addition, previous studies reported that some histone modifications are also involved in the regulation of DNA methylation (Cedar and Bergman 2009; Wang et al. 2019).

We first downloaded the DNA methylase, DNA demethylase, and histone modification protein sequences of *Arabidopsis* and *C. sinensis* (var. *sinensis* cv. 'LJ43') from publications by Wang et al. (Wang et al. 2019) and Tong et al. (Tong et al. 2021). We then utilized these sequences to identify candidate DNA methylase and demethylase sequences in the TGY genome through the BlastP function (E value < e-20) and KEGG annotations (Kong et al. 2023) and further filtered candidate sequences on the basis of the analysis of conserved motifs from Pfam (http://pfam.xfam.org/search/sequence) and SMART (<http://smart.embl-heidelberg.de/>).

Through RNA-seq analysis, we identified only 22 genes (five demethylase genes, ten methyltransferase genes, and seven histone modification genes) whose average expression level was > 1 FPKM in each sample. To determine the cause of the increase in genome-wide weighted methylation under salt and alkali stress, we further analyzed the changes in the expression of these 22 genes under stress.

**Quantitative real-time PCR assays**

To verify the gene expression changes induced by changes in methylation, we performed qRT‒PCR analysis of several important genes (primers in **Table S5**). We extracted total RNA from the samples using the FastPure Universal Plant Total RNA Isolation Kit (RC411-01) (Vazyme Biotech Co., Ltd., Nanjing, China) and reverse transcribed the RNA to cDNA using the PrimeScript RT Reagent Kit (RR037A) (Takara, Japan). The qRT‒PCR mixture (10 μl) was prepared with 2x SYBR Green qPCR Master Mix (US Everbright® Inc. in Suzhou, China). All qRT‒PCR analyses were performed using the CFX96 Touch™ Real-Time PCR Detection System (Bio-Rad, Hercules, CA, USA). The tea plant *Actin* and *GAPDH* genes were employed as internal controls across diverse samples for qRT–PCR (**Table S5**). Fold changes in gene expression were calculated utilizing the 2^−ΔΔCT^ method on the basis of data collected from three independent biological replicates.

**Reference:**

Akalin, A., Kormaksson, M., Li, S., Garrett-Bakelman, F.E., Figueroa, M.E., Melnick, A., and Mason, C.E. methylKit: a comprehensive R package for the analysis of genome-wide DNA methylation profiles. Genome Biology. (2012);**13**(10):R87.

Cao, H., Yue, C., Luo, L., Wang, H., Shao, H., Wu, F., He, L., Lucini, L., and Zeng, L. Muti-omics analysis reveals the anthocyanin biosynthesis and accumulation mechanism in the hawk tea tree (*Litsea coreana* var. *lanuginose*). Food Bioscience. (2024);**62**:105497.

Cedar, H., and Bergman, Y. Linking DNA methylation and histone modification: patterns and paradigms. Nature Reviews Genetics. (2009);**10**(5):295-304.

Chen, C.J., Chen, H., Zhang, Y., Thomas, H.R., Frank, M.H., He, Y.H., and Xia, R. TBtools: An integrative toolkit developed for interactive analyses of big biological data. Molecular Plant. (2020);**13**(8):1194-1202.

Chen, S.F., Zhou, Y.Q., Chen, Y.R., and Gu, J. fastp: an ultra-fast all-in-one FASTQ preprocessor. Bioinformatics. (2018);**34**(17):884-890.

Guo, H., Zhu, P., Yan, L., Li, R., Hu, B., Lian, Y., Yan, J., Ren, X., Lin, S., Li, J., et al. The DNA methylation landscape of human early embryos. Nature. (2014);**511**(7511):606-610.

Kim, D., Landmead, B., and Salzberg, S.L. HISAT: A fast spliced aligner with low memory requirements. Nature Methods. (2015);**12**(4):357-U121.

Kong, W., Zhu, Q., Zhang, Q., Zhu, Y., Yang, J., Chai, K., Lei, W., Jiang, M., Zhang, S., Lin, J., et al. 5mC DNA methylation modification-mediated regulation in tissue functional differentiation and important flavor substance synthesis of tea plant (*Camellia sinensis* L.). Horticulture Research. (2023);**10**(8):uhad126.

Krueger, F., and Andrews, S.R. Bismark: a flexible aligner and methylation caller for Bisulfite-Seq applications. Bioinformatics. (2011);**27**(11):1571-1572.

Law, J.A., and Jacobsen, S.E. Establishing, maintaining and modifying DNA methylation patterns in plants and animals. Nature Reviews Genetics. (2010);**11**(3):204-220.

Li, H., and Durbin, R. Fast and accurate short read alignment with Burrows-Wheeler transform. Bioinformatics. (2009);**25**(14):1754-1760.

Liao, Y., Smyth, G.K., and Shi, W. featureCounts: An efficient general purpose program for assigning sequence reads to genomic features. Bioinformatics. (2014);**30**(7):923-930.

Matzke, M.A., Kanno, T., and Matzke, A.J.M. (2015). RNA-Directed DNA Methylation: The Evolution of a Complex Epigenetic Pathway in Flowering Plants. In: Annual Review of Plant Biology, Vol 66--Merchant, S.S., ed. 243-267.

McKenna, A., Hanna, M., Banks, E., Sivachenko, A., Cibulskis, K., Kernytsky, A., Garimella, K., Altshuler, D., Gabriel, S., Daly, M., et al. The Genome Analysis Toolkit: a MapReduce framework for analyzing next-generation DNA sequencing data. Genome Research. (2010);**20**(9):1297-1303.

Tong, W., Li, R.P., Huang, J., Zhao, H.J., Ge, R.H., Wu, Q., Mallano, A.I., Wang, Y.L., Li, F.D., Deng, W.W., et al. Divergent DNA methylation contributes to duplicated gene evolution and chilling response in tea plants. the Plant Journal. (2021);**106**(5):1312-1327.

Wan, S., Zhang, Y., Liu, L., Xiao, Y., He, J., Zhang, Y., Wang, W., and Yu, Y. Comparative effects of salt and alkali stress on photosynthesis and antioxidant system in tea plant (*Camellia sinensis*). Plant Growth Regulation. (2024);**103**(3):565-579.

Wang, L., Shi, Y., Chang, X.J., Jing, S.L., Zhang, Q.J., You, C.J., Yuan, H.Y., and Wang, H.F. DNA methylome analysis provides evidence that the expansion of the tea genome is linked to TE bursts. Plant Biotechnology Journal. (2019);**17**(4):826-835.

Zhang, Q., Ye, Z., Wang, Y., Zhang, X., and Kong, W. Haplotype-resolution transcriptome analysis reveals important responsive gene modules and allele-specific expression contributions under continuous salt and drought in *Camellia sinensis*. Genes. (2023);**14**(7):1417.

Zhang, X.T., Chen, S., Shi, L.Q., Gong, D.P., Zhang, S.C., Zhao, Q., Zhan, D.L., Vasseur, L., Wang, Y.B., Yu, J.X., et al. Haplotype-resolved genome assembly provides insights into evolutionary history of the tea plant *Camellia sinensis*. Nature Genetics. (2021);**53**(8):1250–1259.

Zhou, Q., Lim, J.-Q., Sung, W.-K., and Li, G. An integrated package for bisulfite DNA methylation data analysis with Indel-sensitive mapping. BMC Bioinformatics. (2019);**20**(1):47.
