# Supplementary material for: Dynamic DNA methylation in tea plants and its association with changes in gene expression under salt and alkali stress
Source: Mol Hortic. 2026 Feb 5;6:10. doi: 10.1186/s43897-025-00189-5 (PMC12874681; doi:10.1186/s43897-025-00189-5)
Supplement: Supplementary file 2 — Supplementary Material 2. Supplementary Figures. [file 43897_2025_189_MOESM2_ESM.docx]

**Dynamic DNA methylation in tea** **plants and its association with changes in gene expression under salt and alkali stress**

Xiangrui Kong ^1+^, Hongli Cao ^2+^, Dandan Lou ^3^, Chuan Yue ^2^, Ruiyang Shan ^1^, Shiqin Zheng ^1^, Aodi Han ^1^, Xingtan Zhang ^4*^, Changsong Chen ^1*^, and Weilong Kong ^4*^

**^1^** Tea Research Institute, Fujian Academy of Agricultural Sciences, Fuzhou, Fujian 350013, China;

**^2^** Integrative Science Center of Germplasm Creation in Western China (CHONGQING) Science City, College of Food Science, Southwest University, Chongqing, China;

**^3^** Key Laboratory of Horticultural Plant Biology, Ministry of Education, National Key Laboratory for Germplasm Innovation & Utilization of Horticultural Crops, College of Horticulture and Forestry Sciences, Huazhong Agricultural University, Wuhan 430070, China;

**^4^** National Key Laboratory for Tropical Crop Breeding, Shenzhen Branch, Guangdong Laboratory for Lingnan Modern Agriculture, Genome Analysis Laboratory of the Ministry of Agriculture, Agricultural Genomics Institute at Shenzhen, Chinese Academy of Agricultural Sciences, Shenzhen, Guangzhou 518120, China;

**Corresponding author**,

Email:

[zhangxingtan@caas.cn](mailto:zhangxingtan@caas.cn);

[ccs6536597@163.com](mailto:ccs6536597@163.com);

Weilong.Kong@whu.edu.cn

**Supplemental figure legends**


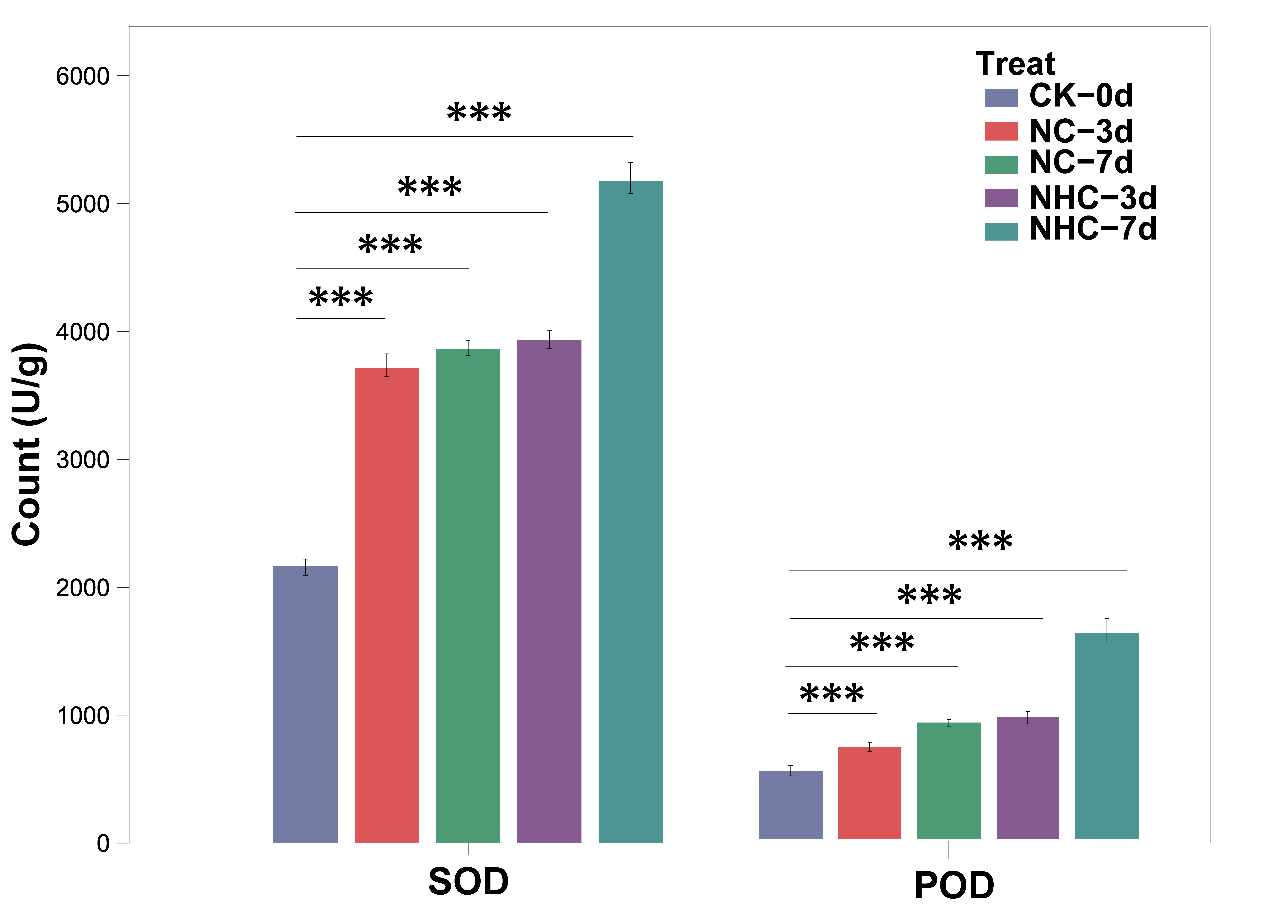


**Figure S1.** Changes in peroxidase (POD) and superoxide dismutase (SOD) contents in young leaves under salt and alkali stress. *** indicates p < 0.001 (paired t test).


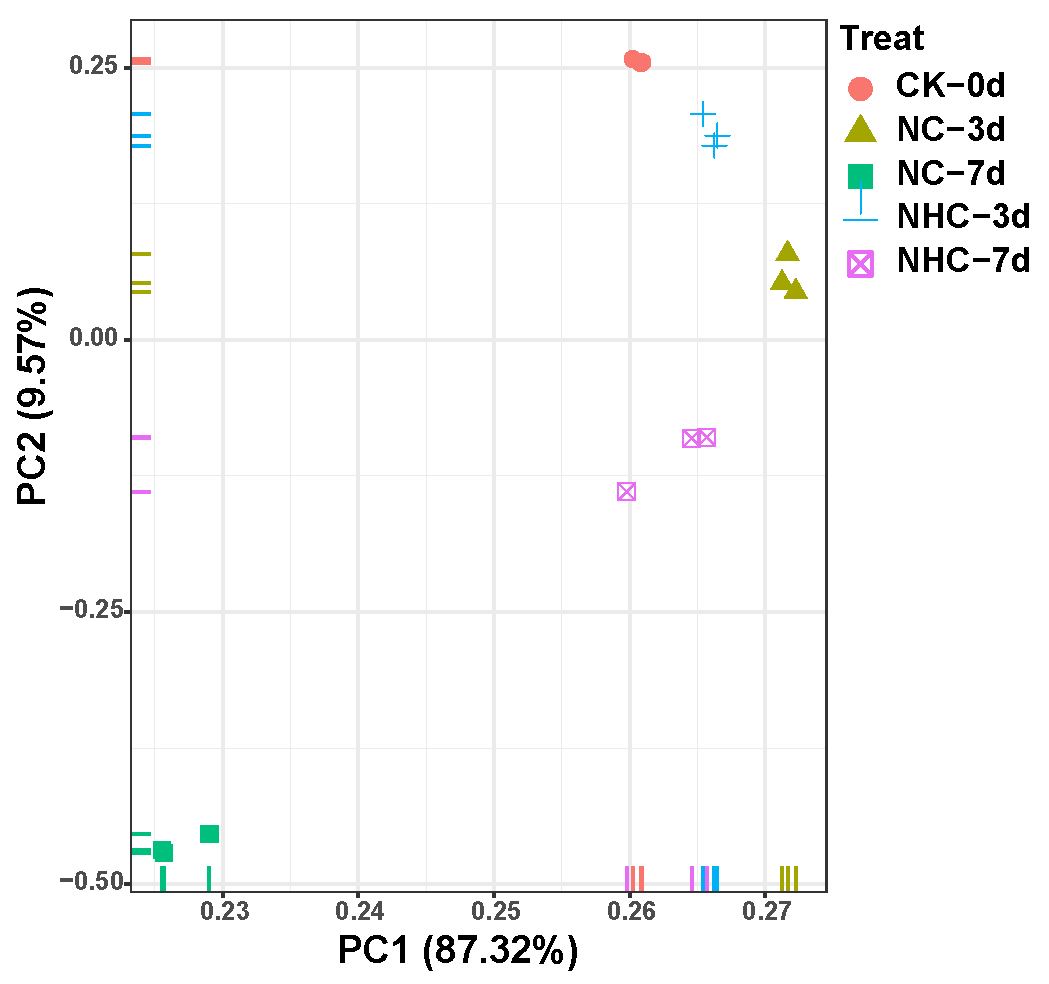


**Figure S2.** Principal component analysis (PCA) of RNA-seq data from different treatment samples.


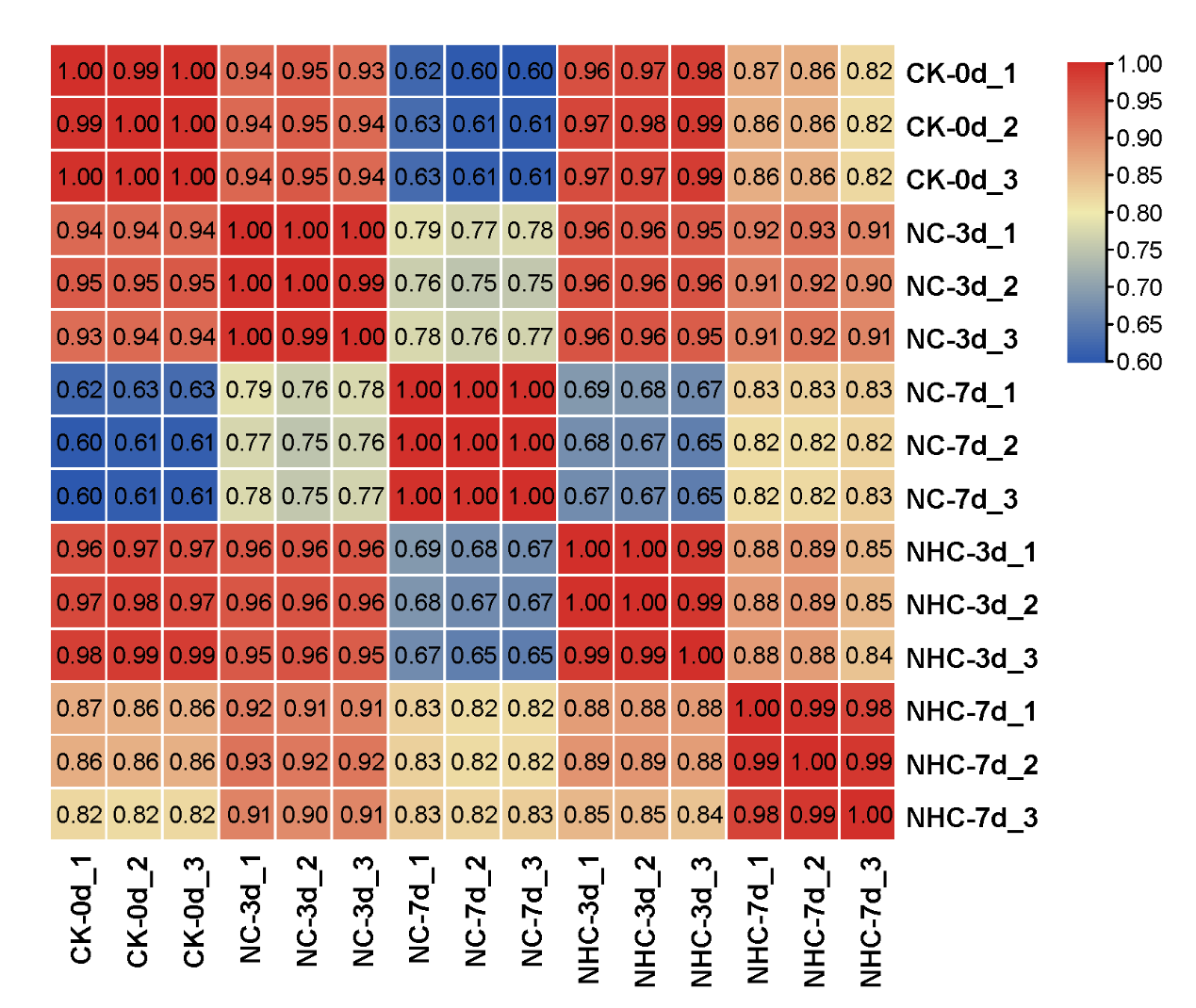


**Figure S3.** The Pearson correlation results for the 15 RNA-seq samples in this study.


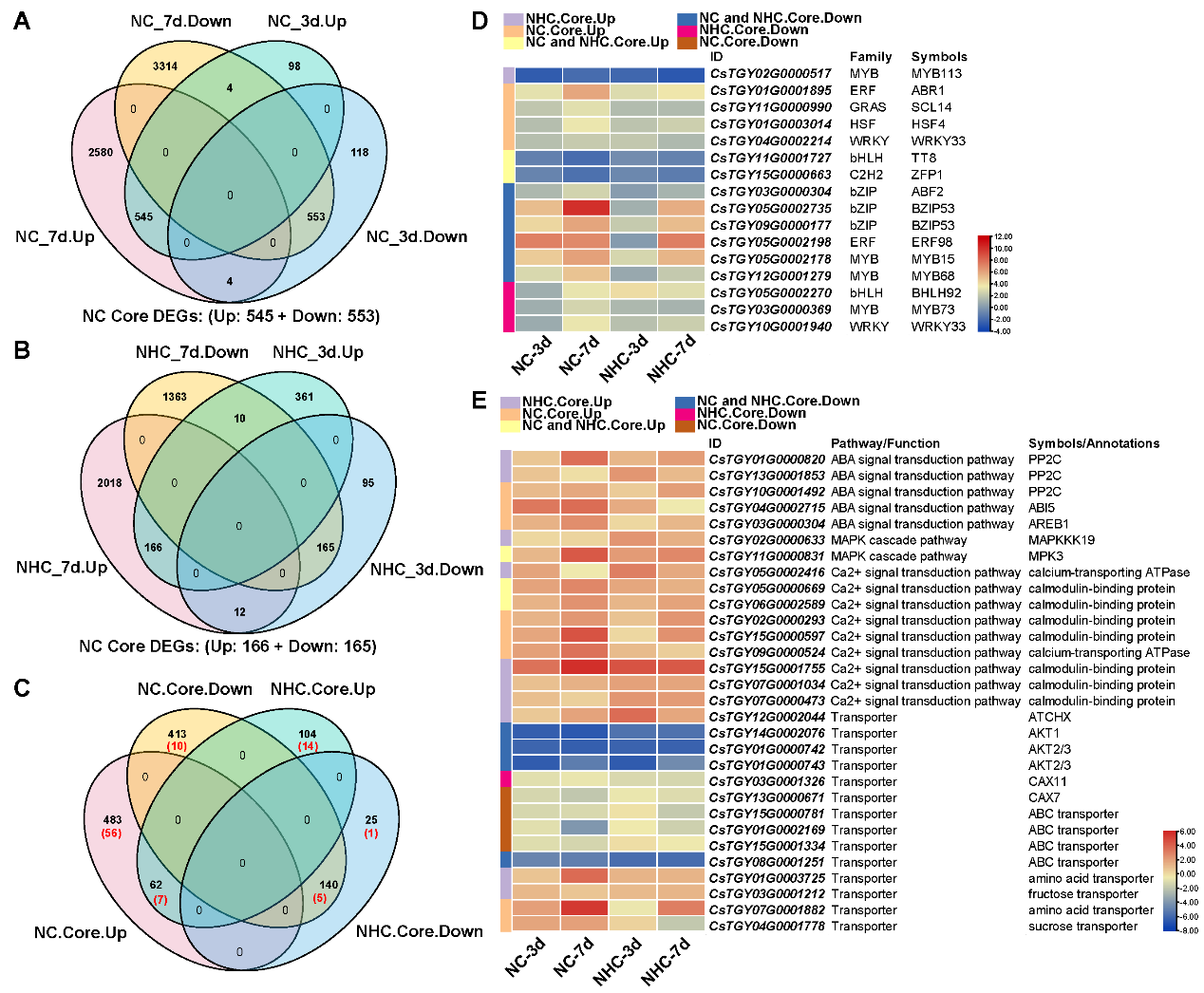


**Figure S4. Changes in the expression of important genes related to transcription factors, signaling pathways, and transporter proteins under salt and alkali stresses.**

**A.** Venn diagrams of DEGs under NaCl treatment (NC).

**B.** Venn diagrams of DEGs under NaHCO_3_ treatment (NHC).

**C.** Venn diagrams of NC and NHC core DEGs.

**D.** Gene expression levels of TF-encoding core DEGs under NC and NHC treatment.

**E.** Gene expression levels of core DEGs related to the ABA signal transduction pathway, MAPK cascade pathway, Ca^2+^ signal transduction pathway, and transporter proteins under NC and NHC treatment.


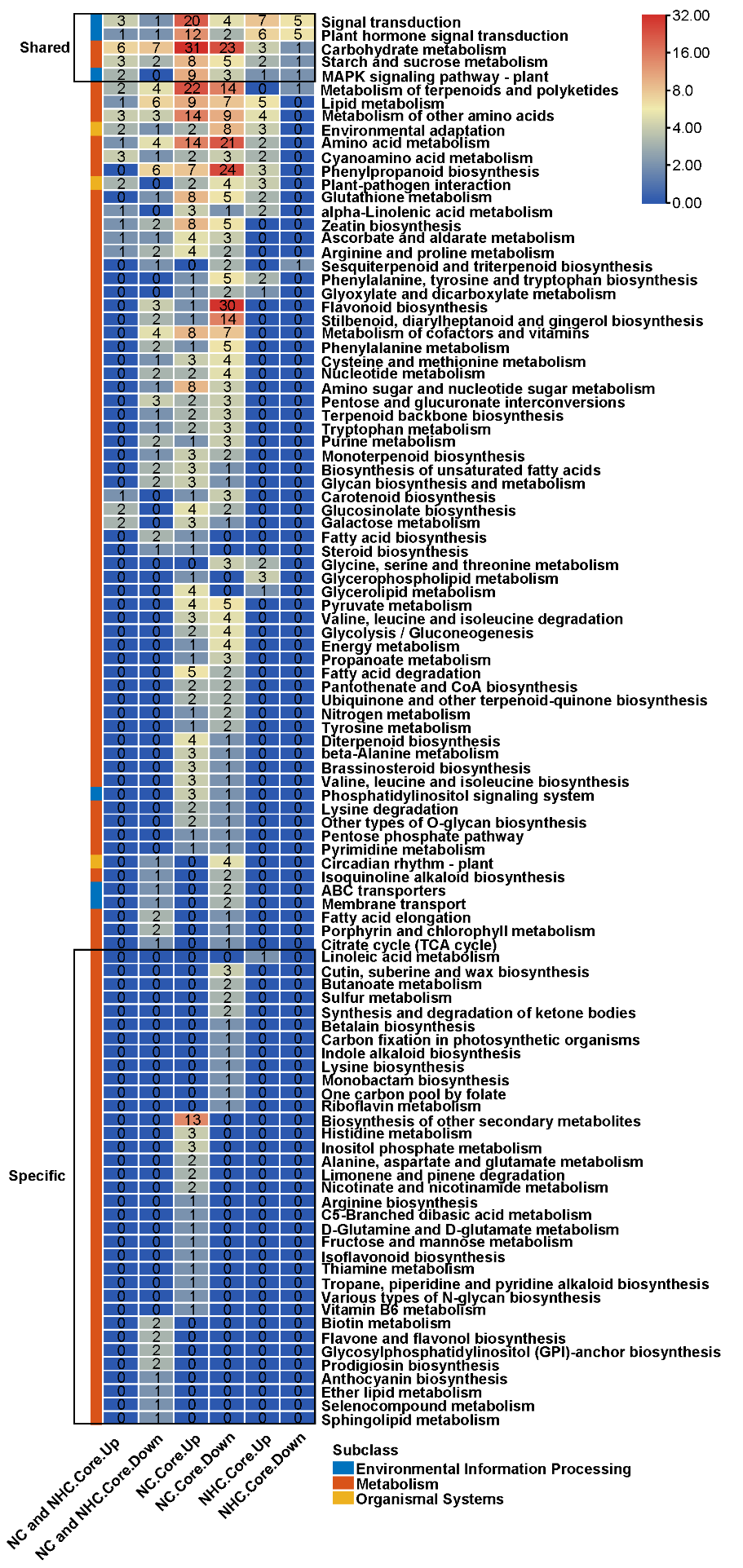


**Figure S5.** Core DEGs in KEGG pathways belonging to the organismal systems, metabolism, and environmental information processing subclasses.


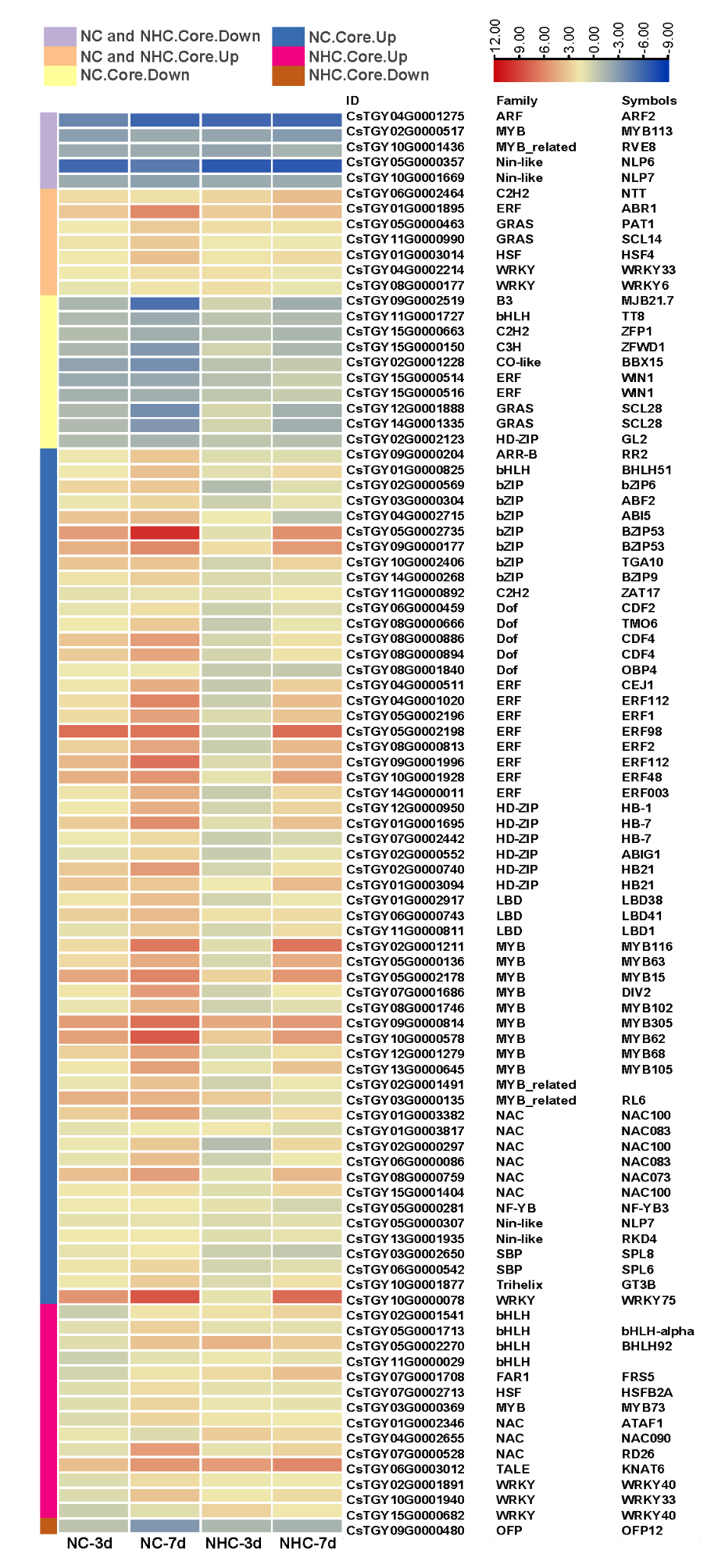


**Figure S6.** Transcription factor-encoding genes in the six core DEG datasets.


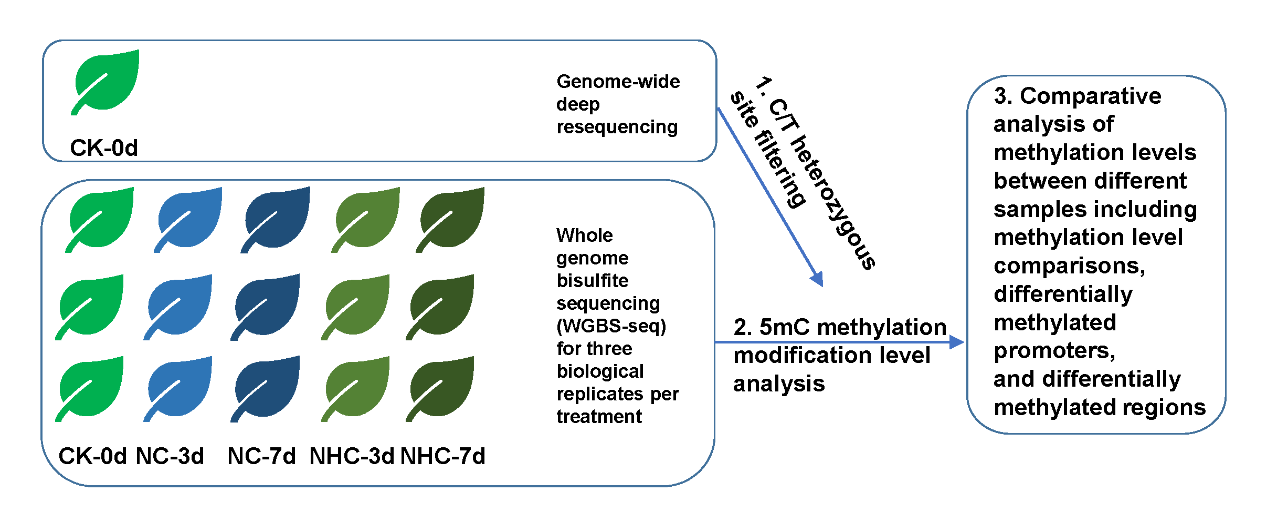


**Figure S7.** WGBS-seq sequencing analysis protocol used in this study.


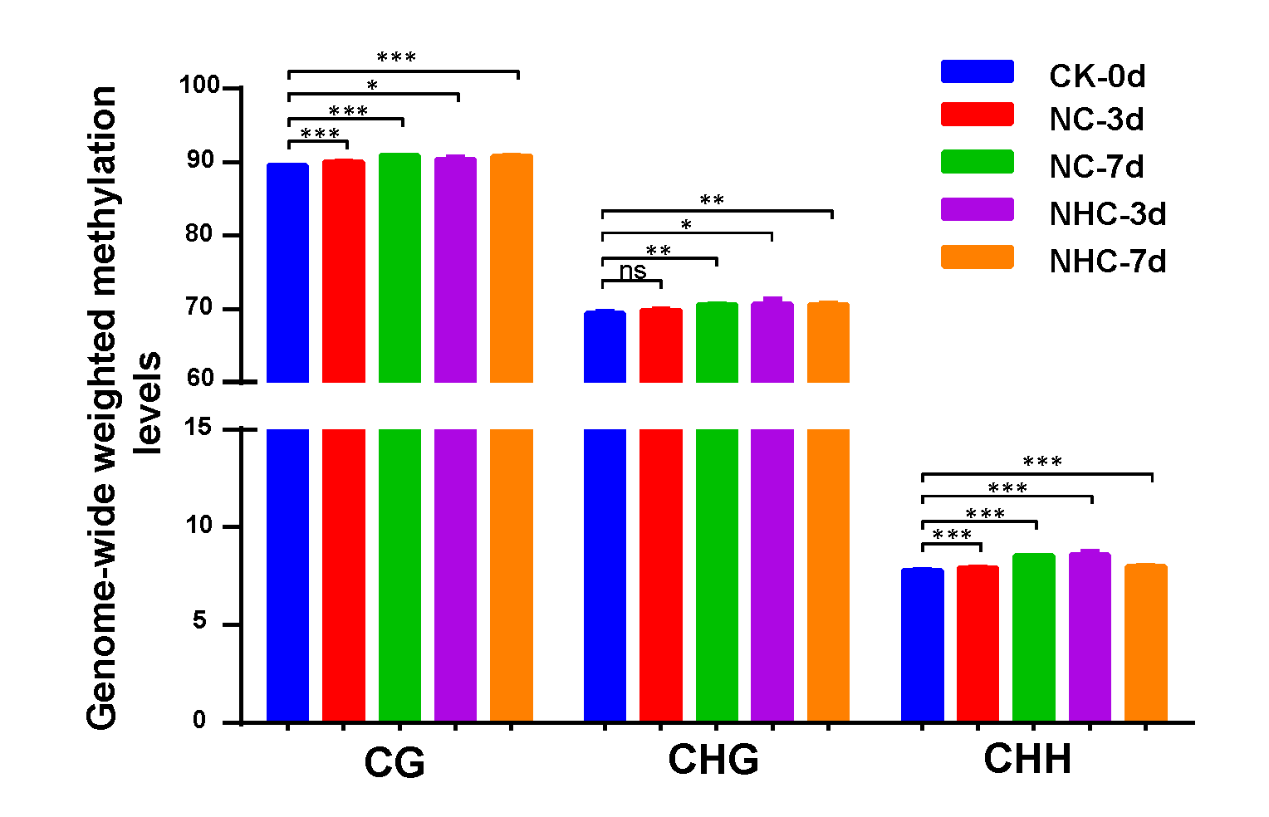


**Figure S8.** Genome-wide weighted methylation levels of samples from different treatment groups. ns, *, **, and *** indicate p > 0.05, p < 0.05, p < 0.01, and p < 0.001 (paired t test), respectively.


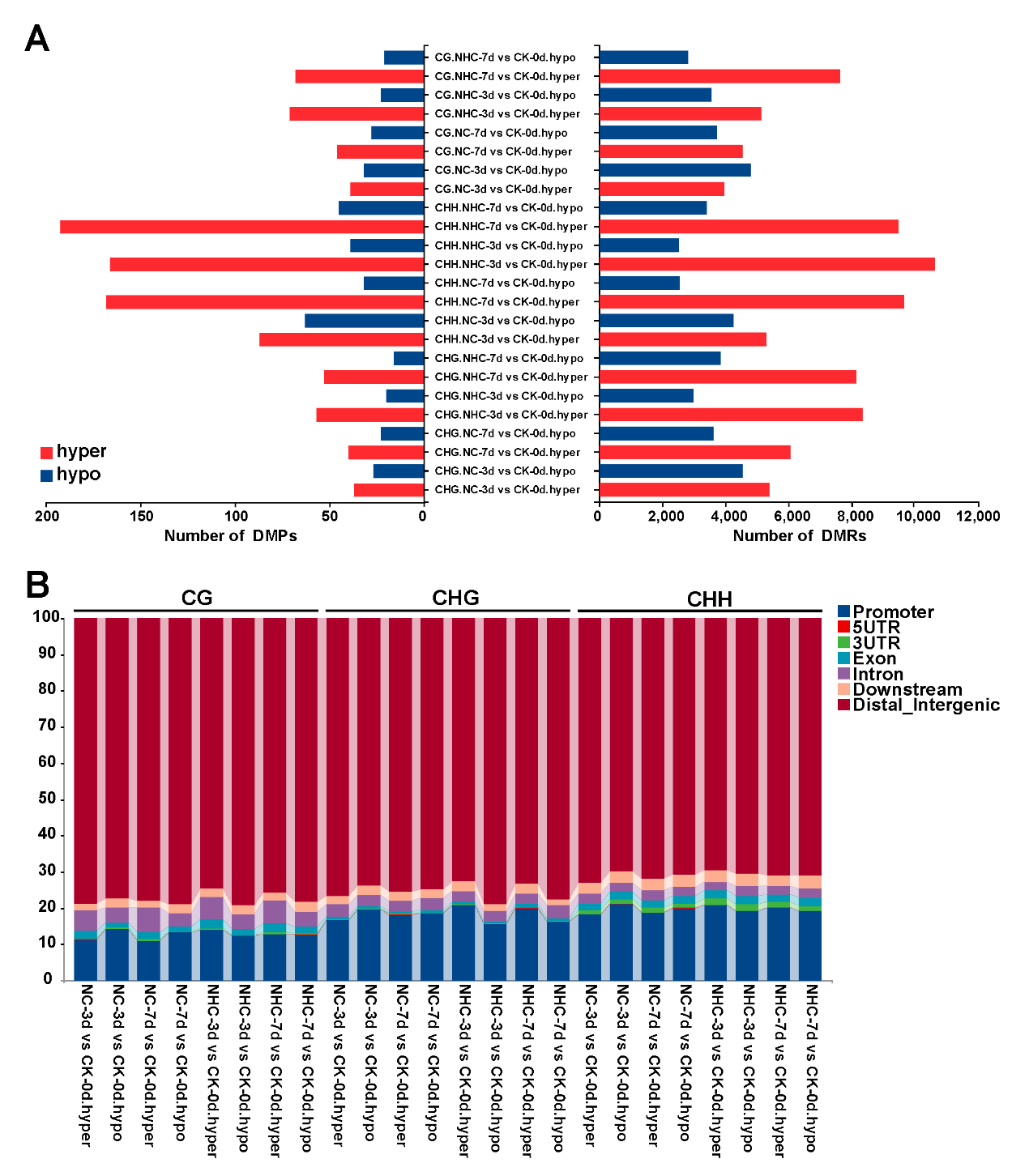


**Figure S9. A.** Number of differentially methylated promoters (DMPs) and differentially methylated regions (DMRs) in samples at different time points under salt and alkali stresses relative to untreated samples. Hyper and Hypo represent hypermethylated and hypomethylated DMRs/DMPs, respectively.

**B.** Genomic position annotation of differentially methylated regions (DMRs).


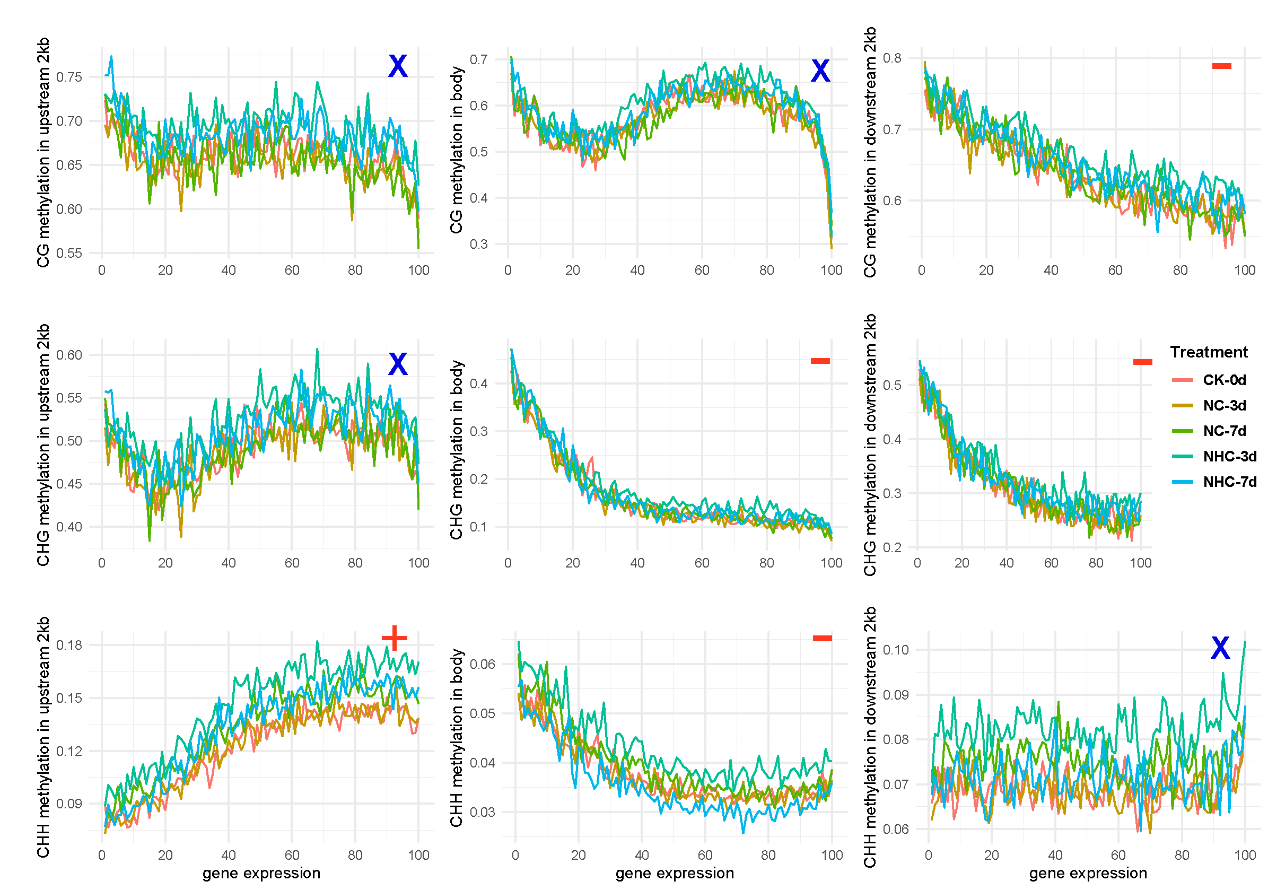


**Figure S10. Methylation profiles of genes with different expression levels.**

Genes were grouped into 100 subgroups (x-axis) according to expression level from low to high. The y-axis shows the methylation levels in 2 Kb upstream, in gene body, and in 2 Kb downstream region for mCG; in 2 Kb upstream, in gene body, and in 2 Kb downstream region for mCHG; and in 2 Kb upstream, in gene body, and in 2 Kb downstream region for mCHH.


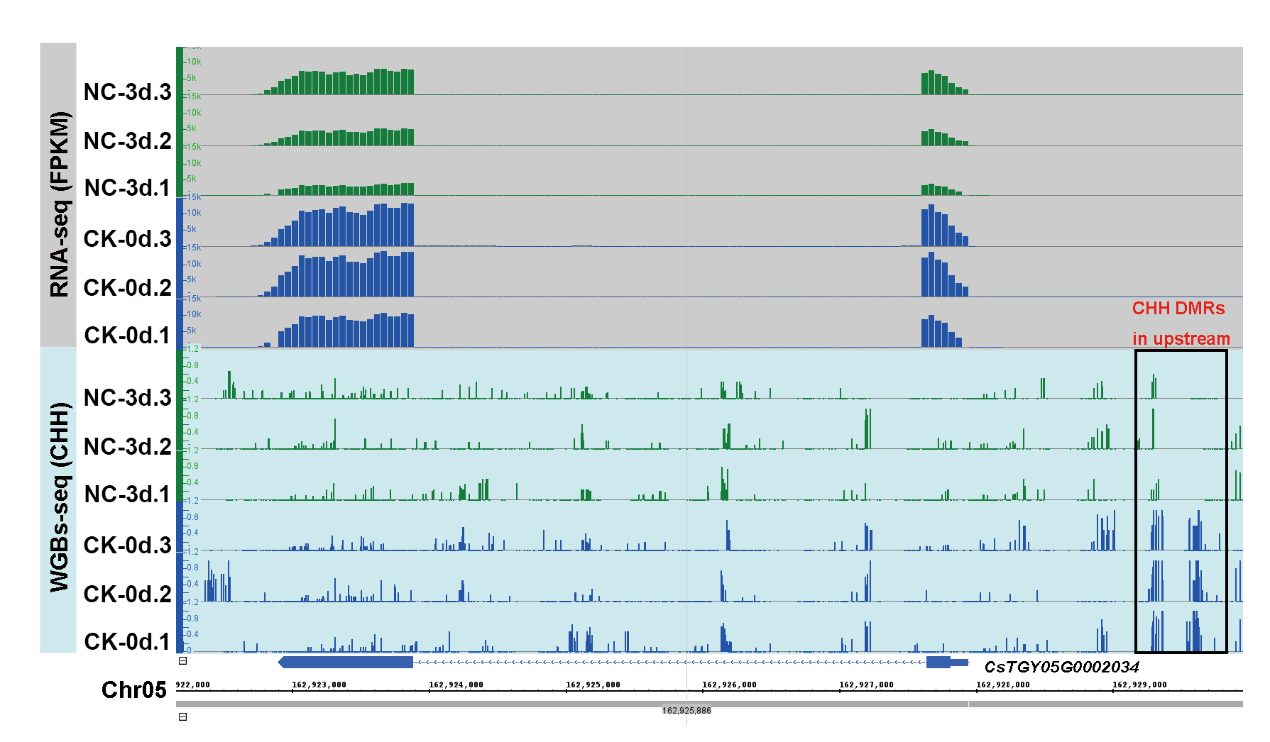


**Figure S11.** Visualization of the methylation and gene expression levels of *CsTGY05G0002034* (*CHS*) under NC-3d.


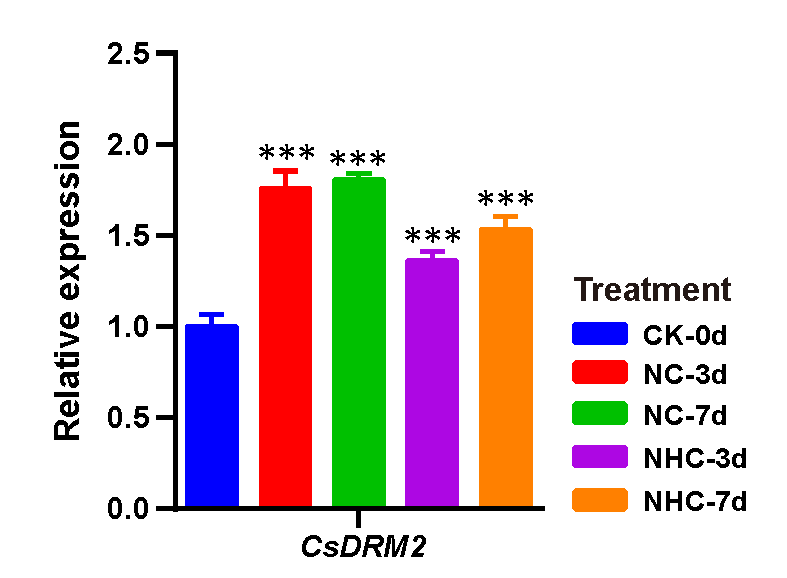


**Figure S12.** qRT‒PCR result for the *CsDRM2* gene under salt and alkali stresses. *** indicates p < 0.001 (paired t test).
